# 2-{[2-(Piperazin-4-ium-1-yl)ethyl­iminio]meth­yl}phenolate 0.06-chloride 0.94-perchlorate

**DOI:** 10.1107/S1600536811042255

**Published:** 2011-10-22

**Authors:** Mohammad Reza Reisi, Hamid Khaledi, Hapipah Mohd Ali

**Affiliations:** a Chemistry Department, Isfahan University 81646-73441, Isfahan, Iran; b Department of Chemistry, University of Malaya, 50603 Kuala Lumpur, Malaysia

## Abstract

The structure of the title salt, C_13_H_20_N_3_O^+^·0.94ClO_4_^−^·0.06Cl^−^, contains a zwitterionic Schiff base with a net positive charge and a perchlorate anion having substitutional disorder with Cl. In the cation, the azomethine N atom is protonated and donates hydrogen bonds to the phenolate O atom and to the tertiary N atom of the piperazine ring. In the crystal, two Schiff base mol­ecules are linked about a center of inversion by a pair of N—H⋯O hydrogen bonds. The resulting dimers are N—H⋯O and C—H⋯O hydrogen bonded to the perchlorate anions, forming a three-dimensional structure. The network is further consolidated by C—H⋯π inter­actions.

## Related literature

For the structure of a nickel(II) complex of the ligand, see: Mukhopadhyay *et al.* (2003 ▶). For the structure of a cadmium(II) complex of the ligand, see: Saleh Salga *et al.* (2010 ▶).
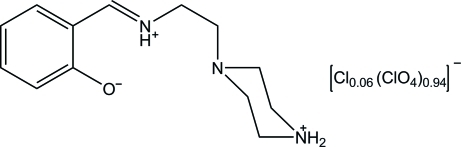


## Experimental

### 

#### Crystal data

C_13_H_20_N_3_O^+^·0.94ClO_4_^−^·0.06Cl^−^
*M* *_r_* = 329.74
Monoclinic, 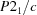

*a* = 11.2322 (2) Å
*b* = 6.5240 (1) Å
*c* = 21.0087 (4) Å
β = 90.597 (1)°
*V* = 1539.41 (5) Å^3^
*Z* = 4
Mo *K*α radiation
μ = 0.27 mm^−1^
*T* = 100 K
0.28 × 0.22 × 0.18 mm

#### Data collection

Bruker APEXII CCD diffractometer
Absorption correction: multi-scan (*SADABS*; Sheldrick, 1996 ▶) *T*_min_ = 0.927, *T*_max_ = 0.952
11909 measured reflections
2860 independent reflections
2393 reflections with *I* > 2σ(*I*)
*R*_int_ = 0.031

#### Refinement

*R*[*F* ^2^ > 2σ(*F* ^2^)] = 0.045
*wR*(*F* ^2^) = 0.127
*S* = 1.04
2860 reflections
213 parameters
6 restraints
H atoms treated by a mixture of independent and constrained refinement
Δρ_max_ = 0.58 e Å^−3^
Δρ_min_ = −0.27 e Å^−3^

### 

Data collection: *APEX2* (Bruker, 2007 ▶); cell refinement: *SAINT* (Bruker, 2007 ▶); data reduction: *SAINT* (Bruker, 2007 ▶); program(s) used to solve structure: *SHELXS97* (Sheldrick, 2008 ▶); program(s) used to refine structure: *SHELXL97* (Sheldrick, 2008 ▶); molecular graphics: *X-SEED* (Barbour, 2001 ▶); software used to prepare material for publication: ’*SHELXL97* (Sheldrick, 2008 ▶) and *publCIF* (Westrip, 2010 ▶)’.

## Supplementary Material

Crystal structure: contains datablock(s) I, global. DOI: 10.1107/S1600536811042255/pv2459sup1.cif

Structure factors: contains datablock(s) I. DOI: 10.1107/S1600536811042255/pv2459Isup2.hkl

Supplementary material file. DOI: 10.1107/S1600536811042255/pv2459Isup3.cml

Additional supplementary materials:  crystallographic information; 3D view; checkCIF report

## Figures and Tables

**Table 1:** Hydrogen-bond geometry (Å, °) *Cg*1 is the centroid of the C1–C6 ring.

*D*—H⋯*A*	*D*—H	H⋯*A*	*D*⋯*A*	*D*—H⋯*A*
N1—H1⋯O1	0.88 (3)	1.92 (3)	2.622 (2)	136 (2)
N1—H1⋯N2	0.88 (3)	2.59 (3)	2.893 (3)	100.9 (19)
N3—H3*A*⋯O4^i^	0.86 (3)	2.23 (3)	3.020 (3)	153 (2)
N3—H3*A*⋯O5^i^	0.86 (3)	2.55 (3)	3.325 (3)	149 (2)
N3—H3*B*⋯O1^ii^	0.96 (3)	1.64 (3)	2.589 (2)	176 (2)
C5—H5⋯O5^iii^	0.95	2.43	3.217 (3)	140
C7—H7⋯O3^iv^	0.95	2.49	3.295 (3)	142
C9—H9*B*⋯O2^v^	0.99	2.47	3.271 (4)	138
C13—H13*A*⋯O5^i^	0.99	2.59	3.423 (4)	142
C13—H13*B*⋯O4^vi^	0.99	2.57	3.263 (3)	127
C3—H3⋯*Cg*1^vii^	0.95	2.70	3.500 (3)	142
C8—H8*A*⋯*Cg*1^v^	0.99	2.99	3.849 (3)	145

Symmetry codes: (i) 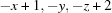; (ii) 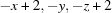; (iii) 
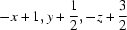
; (iv) 
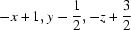
; (v) 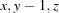; (vi) 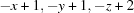; (vii) 
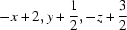
.

**Fig. 1.**
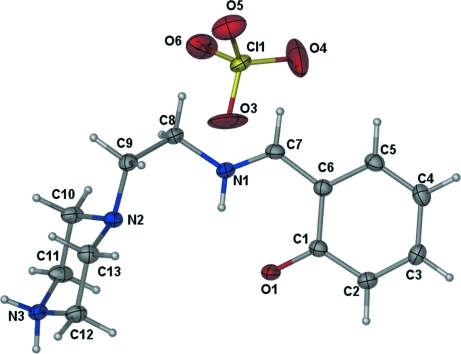
Molecular structure of the title compound with displacement ellipsoids at the 50% probability level. Hydrogen atoms are drawn as spheres of arbitrary radius. The partially occupying choride ion is not depicted.

## supplementary crystallographic information

### Comment

The Schiff base, 1-(2-salicylaldiminoethyl)piperazine, has been shown to exhibit different ligation behavior towards metal ions, mainly depending on the conformation adopted by the piperazine ring (Mukhopadhyay *et al.*, 2003; Saleh Salga *et al.*, 2010). In an attempt to prepare a tin(IV) complex of the Schiff base, the crystals of the title ion-pair were obtained unexpectedly. The Schiff base component is doubly proptonated at its azomethine nitrogen, N1, and its secondary N atoms, N3, while being deprotonated at its oxygen atom, O1. The phenolate O1 atom is hydrogen bond acceptor from the protonated N1 and also from the N3 of a symmetry related molecule, forming a centrosymmetric dimer. The dimers are N—H···O and C—H···O bonded to the perchlorate anions to construct a three-dimensional polymeric structure. The network is further stabilized by C—H···π interactions (Table 1). The anionic part is mainly perchlorate ion which displays small substitutional disorder with Cl [the site-occupancy factor of the perchlorate = 0.937 (2)].

### Experimental

A mixture of salicylaldehyde (0.24 g, 2 mmol) and 4-(2-aminoethyl)piperazine (0.26 g, 2 mmol) in ethanol was refluxed for 2 h. Bu_2_SnCl_2_ (0.6 g, 2 mmol) was then added and reflux was continued for another 2 h. The solution was cooled to room temperature and NaClO_4_ (0.14 g, 1 mmol) was added to the mixture. The precipitaed NaCl was separated out and the filtrate was evaporated under vacumm. The residue was dissolved in dicloromethane and left at room temperature for a day whereupon the brown crystals of the title compound were formed.

### Refinement

The C-bound H atoms were placed at calculated positions and were treated as riding on their parent C atoms with H—C*_sp2_* = 0.95 Å and *H*—*C_methylene_ =* 0.99 Å. The N-bound H atoms were located in a difference Fourier map. For all H atoms, *U*iso(H) was set to 1.2*U*eq(carrier atom). An ISOR restraint (Sheldrick, 2008) was applied to a perchlorate oxygen atom, O3.

### Crystal data

**Table:**

C_13_H_20_N_3_O^+^·0.94ClO_4_^−^·0.06Cl^−^	*F*(000) = 696
*M**_r_* = 329.74	*D*_x_ = 1.423 Mg m^−^^3^
Monoclinic, *P*2_1_/*c*	Mo *K*α radiation, λ = 0.71073 Å
Hall symbol: -P 2ybc	Cell parameters from 3417 reflections
*a* = 11.2322 (2) Å	θ = 2.7–29.0°
*b* = 6.5240 (1) Å	µ = 0.27 mm^−^^1^
*c* = 21.0087 (4) Å	*T* = 100 K
β = 90.597 (1)°	Block, brown
*V* = 1539.41 (5) Å^3^	0.28 × 0.22 × 0.18 mm
*Z* = 4	

### Data collection

**Table:**

Bruker APEXII CCD diffractometer	2860 independent reflections
Radiation source: fine-focus sealed tube	2393 reflections with *I* > 2σ(*I*)
graphite	*R*_int_ = 0.031
φ and ω scans	θ_max_ = 25.5°, θ_min_ = 1.8°
Absorption correction: multi-scan (*SADABS*; Sheldrick, 1996)	*h* = −13→13
*T*_min_ = 0.927, *T*_max_ = 0.952	*k* = −7→7
11909 measured reflections	*l* = −24→25

### Refinement

**Table:**

Refinement on *F*^2^	Primary atom site location: structure-invariant direct methods
Least-squares matrix: full	Secondary atom site location: difference Fourier map
*R*[*F*^2^ > 2σ(*F*^2^)] = 0.045	Hydrogen site location: inferred from neighbouring sites
*wR*(*F*^2^) = 0.127	H atoms treated by a mixture of independent and constrained refinement
*S* = 1.04	*w* = 1/[σ^2^(*F*_o_^2^) + (0.0613*P*)^2^ + 1.4958*P*] where *P* = (*F*_o_^2^ + 2*F*_c_^2^)/3
2860 reflections	(Δ/σ)_max_ = 0.001
213 parameters	Δρ_max_ = 0.58 e Å^−^^3^
6 restraints	Δρ_min_ = −0.27 e Å^−^^3^

### Special details

**Table:**

Geometry. All e.s.d.'s (except the e.s.d. in the dihedral angle between two l.s. planes) are estimated using the full covariance matrix. The cell e.s.d.'s are taken into account individually in the estimation of e.s.d.'s in distances, angles and torsion angles; correlations between e.s.d.'s in cell parameters are only used when they are defined by crystal symmetry. An approximate (isotropic) treatment of cell e.s.d.'s is used for estimating e.s.d.'s involving l.s. planes.
Refinement. Refinement of *F*^2^ against ALL reflections. The weighted *R*-factor *wR* and goodness of fit *S* are based on *F*^2^, conventional *R*-factors *R* are based on *F*, with *F* set to zero for negative *F*^2^. The threshold expression of *F*^2^ > σ(*F*^2^) is used only for calculating *R*-factors(gt) *etc*. and is not relevant to the choice of reflections for refinement. *R*-factors based on *F*^2^ are statistically about twice as large as those based on *F*, and *R*- factors based on ALL data will be even larger.

### Fractional atomic coordinates and isotropic or equivalent isotropic displacement parameters (Å^2^)

**Table:**

	*x*	*y*	*z*	*U*_iso_*/*U*_eq_	Occ. (<1)
O1	0.92876 (14)	0.3289 (3)	0.88619 (8)	0.0288 (4)	
N1	0.72796 (16)	0.1530 (3)	0.85235 (9)	0.0241 (4)	
H1	0.793 (2)	0.153 (4)	0.8761 (12)	0.029*	
N2	0.72451 (16)	−0.0667 (3)	0.97192 (9)	0.0227 (4)	
N3	0.85668 (19)	−0.2651 (3)	1.07210 (10)	0.0279 (5)	
H3A	0.802 (3)	−0.323 (4)	1.0941 (13)	0.033*	
H3B	0.934 (2)	−0.290 (4)	1.0894 (12)	0.033*	
C1	0.90467 (19)	0.4724 (4)	0.84465 (10)	0.0232 (5)	
C2	0.9791 (2)	0.6457 (4)	0.83660 (11)	0.0258 (5)	
H2	1.0491	0.6584	0.8620	0.031*	
C3	0.9523 (2)	0.7957 (4)	0.79292 (11)	0.0265 (5)	
H3	1.0041	0.9099	0.7888	0.032*	
C4	0.8498 (2)	0.7835 (4)	0.75421 (11)	0.0285 (5)	
H4	0.8325	0.8877	0.7239	0.034*	
C5	0.7754 (2)	0.6190 (4)	0.76094 (11)	0.0258 (5)	
H5	0.7057	0.6100	0.7351	0.031*	
C6	0.80000 (19)	0.4627 (3)	0.80549 (10)	0.0224 (5)	
C7	0.71557 (19)	0.3007 (4)	0.81201 (10)	0.0233 (5)	
H7	0.6468	0.3017	0.7853	0.028*	
C8	0.6394 (2)	−0.0046 (4)	0.86529 (11)	0.0267 (5)	
H8A	0.6691	−0.1401	0.8514	0.032*	
H8B	0.5648	0.0255	0.8416	0.032*	
C9	0.61617 (19)	−0.0068 (4)	0.93633 (11)	0.0253 (5)	
H9A	0.5904	0.1311	0.9502	0.030*	
H9B	0.5513	−0.1047	0.9456	0.030*	
C10	0.7358 (2)	−0.2908 (4)	0.97354 (12)	0.0279 (5)	
H10A	0.7346	−0.3456	0.9296	0.034*	
H10B	0.6678	−0.3507	0.9967	0.034*	
C11	0.8518 (2)	−0.3499 (4)	1.00654 (12)	0.0317 (6)	
H11A	0.8584	−0.5011	1.0083	0.038*	
H11B	0.9197	−0.2968	0.9818	0.038*	
C12	0.8353 (2)	−0.0404 (4)	1.07244 (12)	0.0284 (5)	
H12A	0.9028	0.0304	1.0520	0.034*	
H12B	0.8303	0.0085	1.1169	0.034*	
C13	0.7209 (2)	0.0115 (4)	1.03721 (11)	0.0274 (5)	
H13A	0.6525	−0.0499	1.0597	0.033*	
H13B	0.7099	0.1620	1.0365	0.033*	
Cl1	0.40865 (5)	0.51801 (10)	0.85512 (4)	0.0300 (2)	0.937 (3)
O2	0.4847 (3)	0.5543 (5)	0.90856 (15)	0.0754 (9)	0.937 (3)
O3	0.4569 (2)	0.6021 (6)	0.79982 (15)	0.0915 (11)	0.937 (3)
O4	0.29220 (18)	0.5955 (4)	0.86711 (11)	0.0508 (6)	0.937 (3)
O5	0.3970 (2)	0.3003 (3)	0.84855 (11)	0.0556 (7)	0.937 (3)
Cl2	0.4427 (11)	0.4812 (19)	0.8918 (7)	0.035 (4)*	0.063 (3)

### Atomic displacement parameters (Å^2^)

**Table:**

	*U*^11^	*U*^22^	*U*^33^	*U*^12^	*U*^13^	*U*^23^
O1	0.0230 (8)	0.0290 (9)	0.0341 (9)	−0.0034 (7)	−0.0090 (7)	0.0078 (7)
N1	0.0182 (9)	0.0287 (11)	0.0252 (10)	−0.0029 (8)	−0.0033 (8)	0.0015 (8)
N2	0.0191 (9)	0.0236 (10)	0.0254 (10)	0.0013 (8)	−0.0021 (7)	−0.0009 (8)
N3	0.0203 (10)	0.0295 (11)	0.0337 (11)	−0.0054 (8)	−0.0065 (8)	0.0061 (9)
C1	0.0221 (11)	0.0261 (12)	0.0214 (11)	0.0016 (9)	0.0008 (9)	−0.0008 (9)
C2	0.0220 (11)	0.0283 (12)	0.0272 (12)	−0.0016 (9)	−0.0020 (9)	−0.0022 (10)
C3	0.0262 (12)	0.0237 (12)	0.0298 (12)	−0.0021 (9)	0.0064 (9)	−0.0006 (10)
C4	0.0308 (12)	0.0282 (13)	0.0266 (12)	0.0055 (10)	0.0044 (10)	0.0048 (10)
C5	0.0215 (11)	0.0314 (13)	0.0244 (12)	0.0042 (9)	−0.0012 (9)	0.0009 (10)
C6	0.0205 (11)	0.0247 (12)	0.0222 (11)	0.0018 (9)	0.0008 (9)	−0.0022 (9)
C7	0.0184 (10)	0.0292 (12)	0.0223 (11)	0.0021 (9)	−0.0021 (8)	−0.0012 (9)
C8	0.0220 (11)	0.0292 (13)	0.0289 (12)	−0.0053 (9)	−0.0034 (9)	0.0027 (10)
C9	0.0187 (11)	0.0267 (12)	0.0305 (13)	0.0009 (9)	0.0003 (9)	0.0025 (10)
C10	0.0253 (12)	0.0249 (12)	0.0334 (13)	0.0015 (9)	−0.0079 (10)	−0.0040 (10)
C11	0.0291 (12)	0.0253 (13)	0.0406 (14)	0.0053 (10)	−0.0108 (11)	−0.0044 (11)
C12	0.0259 (12)	0.0278 (13)	0.0315 (13)	−0.0027 (10)	−0.0035 (10)	−0.0037 (10)
C13	0.0260 (12)	0.0270 (12)	0.0293 (13)	0.0024 (9)	−0.0014 (10)	−0.0039 (10)
Cl1	0.0209 (3)	0.0334 (4)	0.0357 (5)	−0.0080 (2)	−0.0022 (3)	−0.0010 (3)
O2	0.0628 (17)	0.0668 (17)	0.095 (2)	−0.0180 (14)	−0.0517 (16)	−0.0201 (16)
O3	0.0471 (15)	0.134 (3)	0.094 (2)	0.0135 (16)	0.0184 (14)	0.080 (2)
O4	0.0304 (11)	0.0582 (14)	0.0639 (15)	0.0050 (10)	0.0055 (10)	−0.0243 (12)
O5	0.0702 (16)	0.0310 (12)	0.0651 (15)	−0.0043 (11)	−0.0238 (12)	−0.0091 (10)

### Geometric parameters (Å, °)

**Table:**

O1—C1	1.306 (3)	C6—C7	1.427 (3)
N1—C7	1.290 (3)	C7—H7	0.9500
N1—C8	1.458 (3)	C8—C9	1.518 (3)
N1—H1	0.88 (3)	C8—H8A	0.9900
N2—C13	1.464 (3)	C8—H8B	0.9900
N2—C10	1.468 (3)	C9—H9A	0.9900
N2—C9	1.474 (3)	C9—H9B	0.9900
N3—C11	1.485 (3)	C10—C11	1.519 (3)
N3—C12	1.485 (3)	C10—H10A	0.9900
N3—H3A	0.86 (3)	C10—H10B	0.9900
N3—H3B	0.96 (3)	C11—H11A	0.9900
C1—C2	1.417 (3)	C11—H11B	0.9900
C1—C6	1.429 (3)	C12—C13	1.514 (3)
C2—C3	1.373 (3)	C12—H12A	0.9900
C2—H2	0.9500	C12—H12B	0.9900
C3—C4	1.405 (3)	C13—H13A	0.9900
C3—H3	0.9500	C13—H13B	0.9900
C4—C5	1.369 (3)	Cl1—O3	1.399 (3)
C4—H4	0.9500	Cl1—O2	1.424 (3)
C5—C6	1.410 (3)	Cl1—O4	1.427 (2)
C5—H5	0.9500	Cl1—O5	1.433 (2)
			
C7—N1—C8	125.5 (2)	H8A—C8—H8B	108.4
C7—N1—H1	117.1 (17)	N2—C9—C8	110.59 (18)
C8—N1—H1	117.3 (17)	N2—C9—H9A	109.5
C13—N2—C10	109.16 (18)	C8—C9—H9A	109.5
C13—N2—C9	110.63 (17)	N2—C9—H9B	109.5
C10—N2—C9	110.26 (17)	C8—C9—H9B	109.5
C11—N3—C12	111.59 (19)	H9A—C9—H9B	108.1
C11—N3—H3A	108.3 (19)	N2—C10—C11	109.68 (19)
C12—N3—H3A	108.3 (18)	N2—C10—H10A	109.7
C11—N3—H3B	108.3 (16)	C11—C10—H10A	109.7
C12—N3—H3B	108.3 (16)	N2—C10—H10B	109.7
H3A—N3—H3B	112 (2)	C11—C10—H10B	109.7
O1—C1—C2	122.2 (2)	H10A—C10—H10B	108.2
O1—C1—C6	121.1 (2)	N3—C11—C10	110.6 (2)
C2—C1—C6	116.7 (2)	N3—C11—H11A	109.5
C3—C2—C1	121.5 (2)	C10—C11—H11A	109.5
C3—C2—H2	119.2	N3—C11—H11B	109.5
C1—C2—H2	119.2	C10—C11—H11B	109.5
C2—C3—C4	121.3 (2)	H11A—C11—H11B	108.1
C2—C3—H3	119.3	N3—C12—C13	110.77 (19)
C4—C3—H3	119.3	N3—C12—H12A	109.5
C5—C4—C3	118.8 (2)	C13—C12—H12A	109.5
C5—C4—H4	120.6	N3—C12—H12B	109.5
C3—C4—H4	120.6	C13—C12—H12B	109.5
C4—C5—C6	121.3 (2)	H12A—C12—H12B	108.1
C4—C5—H5	119.3	N2—C13—C12	110.40 (19)
C6—C5—H5	119.3	N2—C13—H13A	109.6
C5—C6—C7	118.2 (2)	C12—C13—H13A	109.6
C5—C6—C1	120.3 (2)	N2—C13—H13B	109.6
C7—C6—C1	121.4 (2)	C12—C13—H13B	109.6
N1—C7—C6	123.3 (2)	H13A—C13—H13B	108.1
N1—C7—H7	118.4	O3—Cl1—O2	110.8 (2)
C6—C7—H7	118.4	O3—Cl1—O4	111.81 (16)
N1—C8—C9	108.35 (19)	O2—Cl1—O4	110.21 (16)
N1—C8—H8A	110.0	O3—Cl1—O5	110.2 (2)
C9—C8—H8A	110.0	O2—Cl1—O5	107.10 (16)
N1—C8—H8B	110.0	O4—Cl1—O5	106.59 (15)
C9—C8—H8B	110.0		

### Hydrogen-bond geometry (Å, °)

**Table:**

Cg1 is the centroid of the C1–C6 ring.

**Table:**

*D*—H···*A*	*D*—H	H···*A*	*D*···*A*	*D*—H···*A*
N1—H1···O1	0.88 (3)	1.92 (3)	2.622 (2)	136 (2)
N1—H1···N2	0.88 (3)	2.59 (3)	2.893 (3)	100.9 (19)
N3—H3A···O4^i^	0.86 (3)	2.23 (3)	3.020 (3)	153 (2)
N3—H3A···O5^i^	0.86 (3)	2.55 (3)	3.325 (3)	149 (2)
N3—H3B···O1^ii^	0.96 (3)	1.64 (3)	2.589 (2)	176 (2)
C5—H5···O5^iii^	0.95	2.43	3.217 (3)	140.
C7—H7···O3^iv^	0.95	2.49	3.295 (3)	142.
C9—H9B···O2^v^	0.99	2.47	3.271 (4)	138.
C13—H13A···O5^i^	0.99	2.59	3.423 (4)	142.
C13—H13B···O4^vi^	0.99	2.57	3.263 (3)	127.
C3—H3···Cg1^vii^	0.95	2.70	3.500 (3)	142
C8—H8A···Cg1^v^	0.99	2.99	3.849 (3)	145

Symmetry codes: (i) −*x*+1, −*y*, −*z*+2; (ii) −*x*+2, −*y*, −*z*+2; (iii) −*x*+1, *y*+1/2, −*z*+3/2; (iv) −*x*+1, *y*−1/2, −*z*+3/2; (v) *x*, *y*−1, *z*; (vi) −*x*+1, −*y*+1, −*z*+2; (vii) −*x*+2, *y*+1/2, −*z*+3/2.
